# Safety standards for intrahospital transfer of critical care patients

**DOI:** 10.1186/cc12222

**Published:** 2013-03-19

**Authors:** D Ashton-Cleary

**Affiliations:** 1 Royal Cornwall Hospital, Truro, UK

## Introduction

The aim was to assess care of patients during intrahospital transfer. The UK Royal College of Anaesthetists has defined auditable standards for the care of patients and the training of escorting medical and nursing staff in this context [1].

## Methods

Patients in a 27-bed combined general and neurosurgical critical care unit were studied in January 2011 and May 2012. Patients undergoing radiology department imaging or intervention were identified from the electronic imaging library. Records of these transfers were sought in the critical care electronic notes and the standards of documentation graded on a five-point scale (very good, good, average, minimal, absent). Documentation of the grade and training of escorting staff was also sought. Between the two study periods, a transfer safety checklist was introduced.

## Results

A total of 20.9% of 143 patients underwent one or more transfers in January 2011 (40 transfers). In May 2012, 26.4% of 151 patients underwent 57 transfers. In the first period, documentation was graded as minimal (limited to a statement that the patient had left the critical care unit) or absent in 77.5% of transfers. In the 62.5% of patients transferred whilst on invasive ventilation, 88.0% had no documentation by the doctor and in 84.0% it was not known which doctor had escorted the patient. There was only slight improvement in the second period (71.9% minimal or absent documentation, 80.0% no documentation by the doctor, 72.0% not known which doctor escorted). In the documentation available, six severe complications were noted during the second period (including episodes of severe bradycardia, hypotension and pupil dilatation).

## Conclusion

On average our unit conducts nearly two critical care transfers each day. Severe complications seem to complicate at least 10% of these, stressing the risk, need for good care and ongoing training. The intervention made in this audit had little impact on the standard of documentation. However, it has raised the issue within the consciousness of the staff. It is important to identify interventions that have failed to reach a gold standard to provide the impetus to seek other solutions. As a result of this study, the author has devised new hospital protocols and specific training courses to improve standards of transfer medicine locally. The study also identified our portable head CT scanner to have the potential to reduce transfers by 52% and so this has been strongly promoted.
